# Endodontic microsurgery utilizing an autonomous robotic system for the maxillary second molar: a case report

**DOI:** 10.1186/s12903-025-06964-6

**Published:** 2025-10-27

**Authors:** Minting Wan, Lishan Huang, Xiaoxing Li, Siyu Li, Qingsong Wu, Chengji Gong, Yufei Li, Xuechao Yang

**Affiliations:** 1 Department of Endodontics, School and Hospital of Stomatology, Guangdong, Engineering Research Center of Oral Restoration and Reconstruction & Guangzhou, Key Laboratory of Basic and Applied Research of Oral Regenerative Medicine, Guangzhou Medical University, Guangzhou, China; 2 Beijing YakeBot Technology Co., Ltd, No. 616, F Building, Yonghe Plaza, 28# Andingmen Dongdajie, Dongcheng District, Beijing, 100007 China

**Keywords:** Endodontic microsurgery, Autonomous robotics, Second molar, Root-end resection, Osteotomy

## Abstract

**Background:**

Endodontic microsurgery (EMS) is a widely utilized technique for addressing periapical periodontitis that is unresponsive to conventional root canal treatment. Nevertheless, achieving precise root apex location and resection can pose significant challenges for surgeons, particularly in complex anatomical situations. The inherent difficulties in accessing and the restricted visualization typically render EMS infeasible in the second molar region. The autonomous robotic (ATR) system, characterized by its automation, precision, and stability, is anticipated to address the limitations inherent in manual operations within complex EMS scenarios. Herein, this report details the successful application of robot-assisted EMS in a maxillary second molar.

**Case presentation:**

A 26-year-old female patient presented to our hospital with chronic periapical periodontitis following a previous history of root canal treatment in the left maxillary second molar. The patient data were imported into DentalNavi software to design the drilling path for precise resection while avoiding damage to the maxillary sinus. Through the integration of an optical pose tracking mechanism and the computerized operational system, the robotic arm completed the autonomous resection task of the mesiobuccal root according to the preoperative plan, providing a position reference for the freehand operation of the distobuccal root. No complications were reported during the surgery. Clinical and radiographic assessments at a six-month follow-up indicated satisfactory outcomes.

**Conclusions:**

The ATR system offers an accurate, safe, and minimally invasive technique for osteotomy and apicoectomy. This technology demonstrates potential as a reliable and clinically effective technique for managing complex and anatomically challenging posterior EMS procedures.

## Background

The pathogenesis of refractory periapical periodontitis is typically related to external root surface microbial infections and abnormal root canal anatomy, which usually cannot be resolved through conventional root canal treatment (RCT) [1]. With the advancements in cone beam computed tomography (CBCT), microsurgical instruments, and bioceramic materials, modern endodontic microsurgery (EMS) has become a frequently employed intervention for preserving natural teeth following unsuccessful RCT [2, 3]. However, posterior molars present significantly greater surgical challenges than anterior teeth, often resulting in less favorable outcomes [4, 5]. The anatomical positioning of the second molar poses substantial challenges for precise localization and root-end resection due to the limited accessibility of instruments, obstruction by soft tissue, and a restricted visual field, even for experienced surgeons [3, 6, 7]. Additionally, the proximity of the root apex of maxillary molars to the maxillary sinus necessitates careful attention to potential sinus perforation, thereby increasing the technical sensitivity and risk of complications [8].

Static navigation (SN), grounded in the concept of digital-guided therapy, was introduced into EMS [9]. This technique employs a computer-designed, 3D-printed template to guide osteotomy and apicoectomy, resulting in more precise and minimally invasive surgical outcomes than freehand surgery [10]. However, the inherent bulk of the navigation template inevitably limits its application in narrow vestibular fornix with restricted access, particularly concerning the second molars. In contrast, Dynamic navigation (DN), with its optical tracking system, offers real-time visualization and is better suited for managing complex EMS cases [11]. Despite its advantages, DN has limitations, including the high demands on hand-eye coordination, the potential for deviation due to the absence of physical constraints, and the impact of additional hand accessories on operator tremors and fatigue [12]. These factors collectively constrain the efficacy of DN in procedures involving the second molars and increase the risk of iatrogenic injury [7]. The piezoelectric “bone window” technique offers an adequate view of the surgical area in the EMS of mandibular second molars [6]. However, the preparation and repositioning of the bony lid also underscore the importance of clinical experience and skill. Regardless of the technology employed, the critical aspects of a surgical procedure are still executed manually by surgeons, where human factors can influence the accuracy of the surgery. Consequently, further exploration of more promising alternative surgical options for second molars is warranted.

In recent years, surgical robotics has made significant advancements in refinement, intelligence, and autonomy [13]. The autonomous robotic (ATR) system is an advanced technology that has been demonstrated to enhance accuracy and mitigate risks during implant surgeries [14–16]. This technique employs an optical pose tracking mechanism and a computerized operational system to direct the robotic arm in executing autonomous movements and drilling tasks according to the preoperative plan [15]. Owing to the precision and task autonomy of the robotic arm, robot-assisted endodontic microsurgery (RA-EMS) holds promise for increased accuracy and predictability in osteotomy and apicoectomy, potentially minimizing the risk of human error and improving surgical prognosis [17, 18].

Therefore, utilizing autonomous and fatigue-free robotic systems offers a promising alternative to address the limitations inherent in manual operations, particularly in complex anatomical scenarios that require enhanced precision and safety in access procedures. This article presents a case of RA-EMS successfully performed on a maxillary second molar adjacent to the maxillary sinus, with satisfactory healing observed at a six-month follow-up.

## Case presentation

**Fig. 1:**
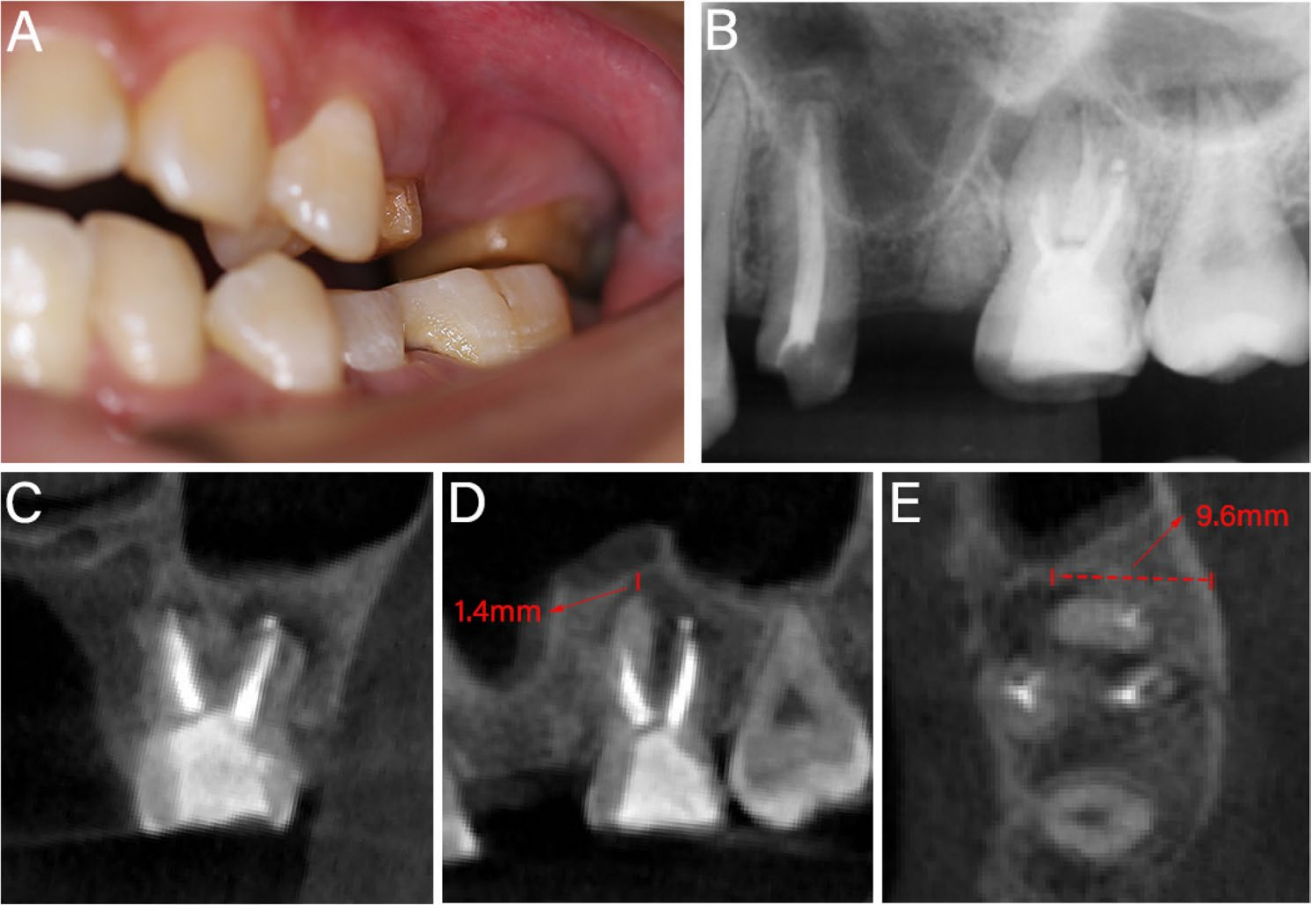
Preoperative examination. **A** Preoperative photograph. **B** Preoperative periapical radiograph. **C** Coronal view of preoperative CBCT. **D** Sagittal view of preoperative CBCT, showing MB apex distance 1.4 mm from the maxillary sinus. **E** Axial view of preoperative CBCT, showing intact buccal cortical plate and the resection depth was 9.6 mm from the MB root's lingual surface to the cortical bone

A 26-year-old female presented at the Affiliated Stomatology Hospital of Guangzhou Medical University, complaining of chewing pain in the left maxillary second molar for two weeks. Teeth #13 and #15 had previously received fixed partial denture restoration, which was later removed, and then underwent RCT of Tooth #15 in our department 7 months ago. During treatment, the second mesiobuccal (MB2) canal and the apical portion of the MB canal were found to be calcified, while the other canals were treated effectively. Clinical examination during this visit revealed that tooth #15 had been crown-prepared and restored with resin (Fig. 1A). Tooth #15 exhibited tenderness to percussion and palpation, no response to pulp sensitivity testing, physiological mobility, normal probing depth, and no sinus tract was observed. Radiological examinations revealed radiolucency associated with the MB and distobuccal (DB) roots. The DB canal, palatal canal, and the upper portion of the MB canal were obturated. However, the MB2 canal and the apical portion of the MB canal were not visible and unfilled (Fig. 1B-E). The apices of tooth #15 were in close proximity to the maxillary sinus, with the minimum distance from the MB apex to the sinus floor measuring 1.4 mm (Fig. 1D). Additionally, the buccal cortical bone plate was intact (Fig. 1E). Tooth #15 was diagnosed with chronic periapical periodontitis following a previous history of RCT. Given the limited efficacy of retreatment options and the insufficiency of a freehand surgical approach as confirmed by preoperative simulation, the patient was recommended to undergo EMS assisted by the ATR system (Yakebot; Yakebot Technology Co., Ltd, Beijing, China). The patient’s informed consent was duly obtained.

### Data acquisition

**Fig. 2:**
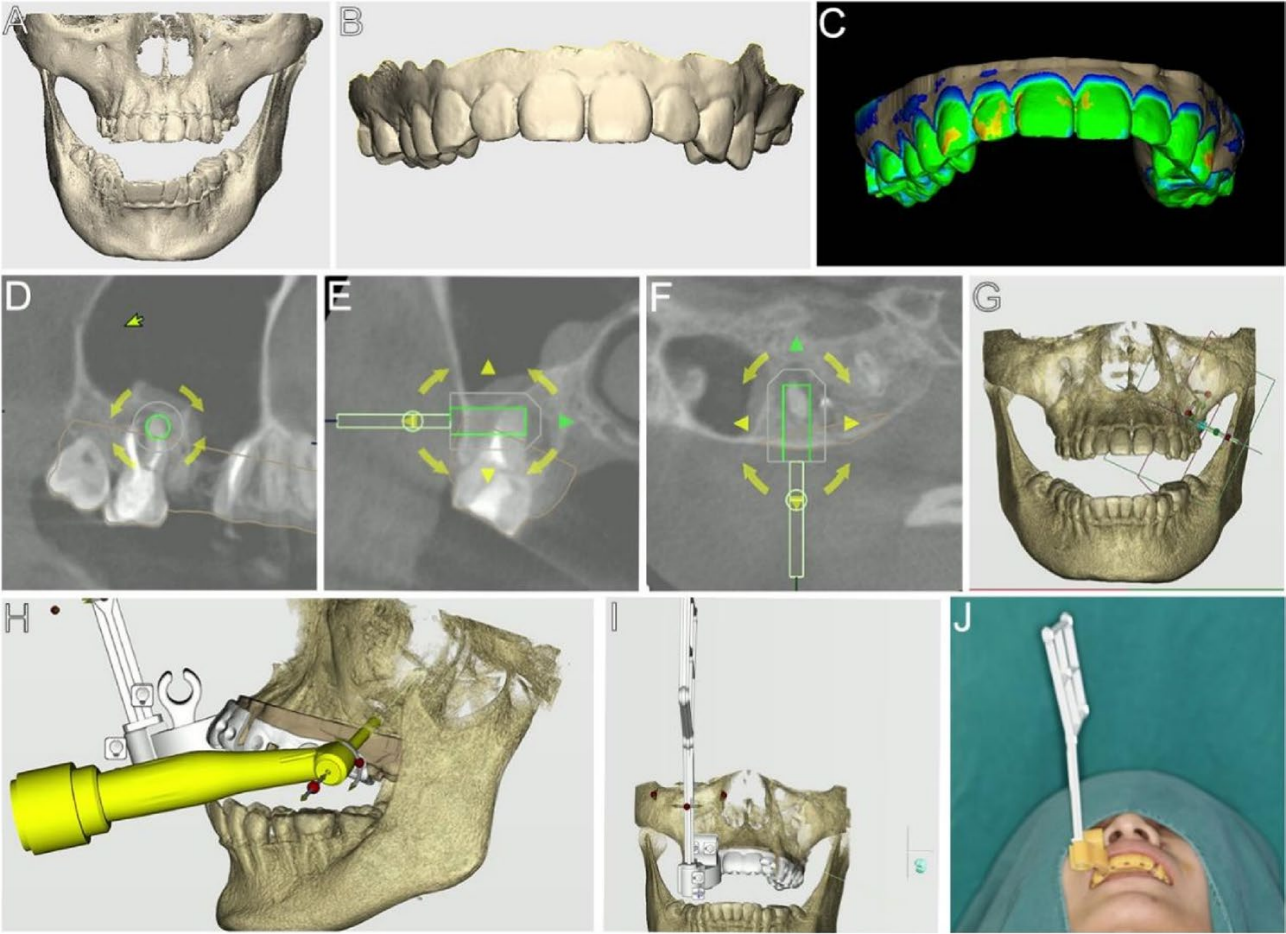
Preoperative preparation. **A** Jaw data obtained by a CBCT scan. **B** Intraoral scan data including teeth and soft tissues. **C** Fit CBCT and intraoral scan data to reconstruct the 3D model. **D**-**H** Design drilling path. **I**-**J** Design and manufacture surgical personalized accessories and visual marker components

The patient’s jaw data were acquired using a CBCT scan (Carestream Health Inc., Rochester, NY, USA) and exported in Digital Imaging and Communications in Medicine format (Fig. 2A). An intra-oral scanner, Cerec Omnicam (Sirona, Bensheim, Germany), was employed to capture the data of the teeth and soft tissues, which were then stored in Standard Tessellation Language format (Fig. 2B).

### Surgical planning

The data were imported into DentalNavi software (Yekebot Technology Co., Ltd, Beijing, China) to reconstruct a 3D model and generate a virtual patient (Fig. 2C). The surgical plan was developed to position a virtual bone trephine bur to resect 3 mm of the MB root-end perpendicularly (Fig. 2D-G). A 3.5 mm diameter, 10 mm length bone trephine bur was selected (Fig. 2H). The drilling path was meticulously designed to avoid the maxillary sinus. Due to limited traction of the cheek mucosa, the DB apex was treated freehand during surgery, using the target point of the MB apex as a reference. Personalized surgical accessories were 3D printed, and the visual marker was designed to be parallel to the patient’s sagittal plane (Fig. 2I and J).

### Surgical procedure

**Fig. 3:**
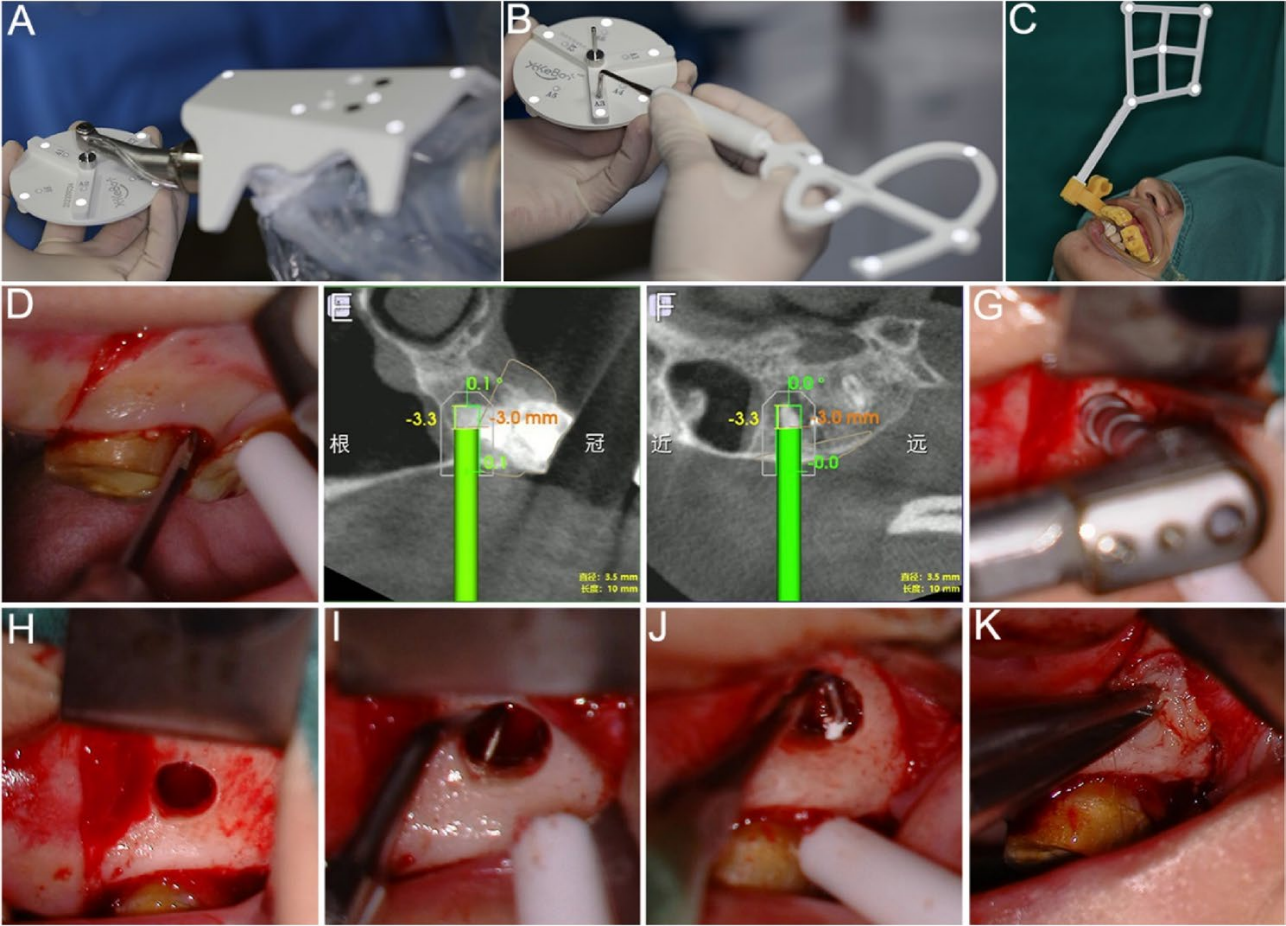
Calibration, registration, and surgical procedure. **A**-**B** Calibration of robotic arm, trephine bur, and probe tip. **C** Intraoral registration. **D** Rectangular flap. **E**-**F** Implementing bone and root-end resection of MB root by the ATR system. **G** Bone and root-end resection of DB root. **H** Resection completed. **I** Retrograde preparation. **J** Retrograde filling. **K** CGF membranes placement

On the surgery day, the robotic instrument was positioned near the surgical site. The extraoral registration was performed under the tracking of infrared optical system. Initially, the robotic arm joint spatial positions were calibrated by tracking the end calibration component. The bur placement point spatial coordinates were then calibrated by attaching the handpiece to the calibration drill mounted on the disc-shaped calibrator. Subsequently, the trephine bur was connected to the handpiece and vertically aligned with the calibrator plane to determine its total length and axial spatial orientation. Finally, the calibration probe was calibrated using the disc calibrator to complete the system setup (Fig. 3A and B).

Anesthesia was administered to the surgical area using 1.7 mL 4% articaine and epinephrine 1/100,000. The surgery was performed by an endodontic specialist with more than 10 years of experience in EMS. A rectangular flap was designed and implemented to ensure full exposure of the surgical area (Fig. 3D).

Subsequently, the surgical accessories equipped with a visual marker were affixed to the anterior dentition of the surgical dental arch. The intraoral registration was completed by using the calibrated probe sequentially connected to six registration holes of the intraoral component (Fig. 3C). Infrared optical recognition of the visual marker enabled the jaw positioning. The manual guided path of the robotic arm was recorded. Ultimately, the robot system transitioned to the autonomous mode to prepare for tissue resection.

The surgeon used the foot pedal to control the autonomous procedure and monitored the process via the DentalNavi interface during entire process. The robotic arm automatically entered the target site, then performed osteotomy and root-end resection with sterile saline irrigation, following the pre-designed path at a rotational speed of 800-1200 rpm (Fig. 3E and F). Upon reaching the preset resection depth, the robotic arm automatically exited to the original position. The ATR system completed the entire autonomous procedure including entry, drilling, and exit in just 80 s.

**Fig. 4:**
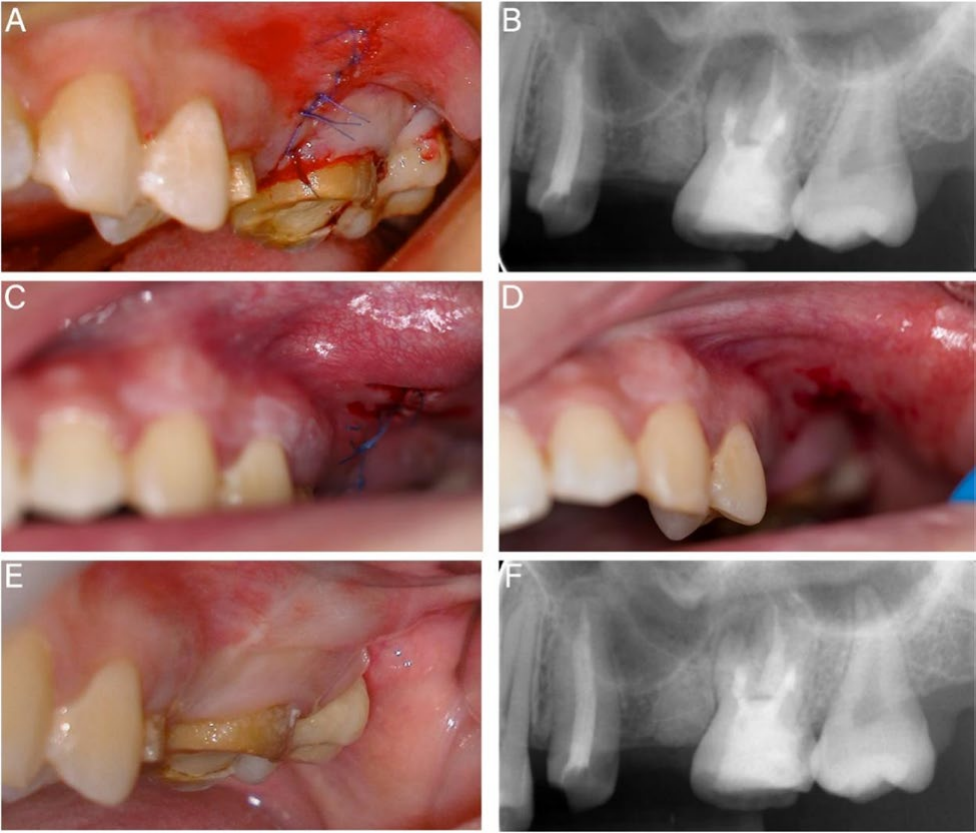
Postoperative examination results. **A**-**B** Immediate postoperative photograph and radiological examination. **C** 1-week follow-up intraoral photograph. **D** Sutures removal. **E**-**F** 6-month follow-up

After resecting the MB root-end, the surgeon enlarged the bone cavity under a microscope (OPMI Proergo; Carl Zeiss, Göttingen, Germany) and resected the DB root-end by 3 mm (Fig. 3G and H). Careful periapical curettage was performed. No maxillary sinus floor injury was observed. The resected root surface showed no cracks, but the MB2 apical foramen was not found. The 3 mm depth of the MB and DB root ends was prepared with an ultrasonic tip (Obtura Spartan, Fenton, MO) and filled with iRoot BP Plus (Innovative Bioceramics, Vancouver, BC, Canada) (Fig. 3I and J). Concentrated growth factors (CGF) were prepared from 20 ml of the patient’s venous blood, pressed into membranes, and placed in the bone cavity (Fig. 3K). The 6 − 0 monofilament sutures were used for flap suturing (Fig. 4A).

### Results

An immediate postoperative radiological examination confirmed the accurate resection of the root ends (Fig. 4B). Sutures were removed a week post-surgery. The patient was asymptomatic and reported taking the analgesic drugs only on the first day. The clinical examination revealed only slight redness and swelling in the soft tissue of the surgical area (Fig. 4C and D). Follow-up evaluations at six months demonstrated satisfactory clinical outcomes, with radiological evidence indicating a recovery in periapical bone density (Fig. 4E and F).

## Discussion

EMS of the second molar poses substantial challenges for clinicians due to its limited accessibility and poor visualization of the apical region, demanding exceptional technical sensitivity [5, 6]. When the buccal cortical bone remains intact, excessive bone resection due to difficulty in apex localization can impair healing and exacerbate postoperative pain [19]. Furthermore, the close anatomical relationship between the maxillary second molar’s apex and the maxillary sinus increases the risk of surgical complications [8, 20–22]. These constraints present significant challenges to the precision, stability, and safety of surgery. Owing to its complex anatomical position and limited treatment options, the second molars are universally regarded as the most challenging targets and are often excluded from EMS intervention.

Dental surgical robotic technology represents an emerging advancement in endodontics, offering transformative potential in the management of pulp and periapical diseases. Robotic technology provides distinct advantages in terms of autonomy, precision, and stability, thereby reducing uncertainty and complexity in clinical surgical interventions [18]. While surgical robotic applications in endodontics are not yet routine, several published studies have explored their effectiveness (Table 1). Recent in vitro studies indicate that the ATR system provides superior accuracy and enhanced time efficiency compared to SN and DN technologies in assisting EMS procedures [17, 23]. Current clinical evidence supports the accuracy of robotic-assisted systems in both EMS [24–27] and RCT [28–31] procedures, encompassing the surgical areas from anterior teeth to the first molars. Our previous study also demonstrated the clinical feasibility of the ATR technique for managing anatomically challenging first molar regions [27]. These reinforce our confidence in tackling more challenging surgical scenarios and further exploring the extended applicability of ATR technology. This report presents the first known case of ATR-assisted EMS for maxillary second molar, achieving accurate, minimally invasive apical resection with a favorable prognosis.

**Table 1:** The summary of literature related to surgical robotic applications in the field of endodontics

Author/year	Study design	Tooth	Robotic system	Endodontics procedure	Clinical purpose	Autonomy level	Follow-up and outcome
Chen C et al. [17], 2024	In vitro study	Maxillary and mandibular jaw models	ATR system	Assist EMS	Compare the accuracy and operation time of ATR, DN, and SN	Active	ATR exhibited the highest accuracy; SN exhibited the shortest operation time, followed by ATR, and then DN
Liu C et al. [23],2024	In vitro study	Mandibular jaw models	ATR system	Assist EMS	Compare the accuracy and treatment time of ATR and DN	Active	ATR showed higher accuracy and shorter drilling time
Isufi A et al. [24], 2024	Case report	Left maxillary first and second premolars	Yomi Robot system	Assist EMS	Osteotomy and root-end resection	Passive	10 days successful
Liu C et al. [25], 2024	Case report	Left mandibular first molar	ATR system	Assist EMS	Osteotomy and root-end resection	Active	1 month successful
Fu M et al. [26], 2025	Case report	Right maxillary lateral incisor	ATR system	Assist EMS	Removal of a fractured file beyond the apical foramen	Active	9 months successful
Huang L et al. [27], 2025	Case series	Four first molars	ATR system	Assist EMS	Osteotomy, root-end resection, and protection of critical anatomical structures	Active	1–6 months successful
Wang J et al. [28], 2024	Case report	Left mandibular first molar	ATR system	Assist RCT	Removal of fiber posts	Active	3 months successful
Qin L et al. [29], 2025	Case report	Left mandibular second premolar	ATR system	Assist RCT	Fiber post removal through a zirconia crown	Active	2.5 months successful
Huang X et al. [30], 2025	Case report	Left maxillary central incisor	ATR system	Assist RCT	Calcified root canal location	Active	9 months successful
Yu P et al. [31], 2025	Case report	Left maxillary central incisor	Remebot system	Assist RCT	Calcified root canal location	Semi-active	12 months successful
Present case	Case report	Left maxillary second molar	ATR system	Assist EMS	Osteotomy, root-end resection, and protection of the maxillary sinus	Active	6 months successful

Under the anatomical challenges of this case, our preoperative simulation by an endodontic specialist demonstrated that it is inadequate for achieving accurate apical location and safe access via the freehand approach. Meanwhile, both the DN and SN technologies were rejected due to their technical limitations in managing buccal root-end in this scenario. The ATR system was consequently implemented to execute precise osteotomy and apicoectomy on maxillary second molar, overcoming several clinical dilemmas that are beyond the capabilities of manual operation. First, the ATR system automates tasks through a robotic platform that integrates infrared optical systems with real-time computer guidance, translating pre-designed data into precise, computer-guided instructions [15]. This technology enables autonomous drilling while providing real-time trajectory feedback through the user interface, ensuring the surgeon’s discrete control over it [14, 32]. These features eliminate the need for direct visual confirmation at the surgical site, effectively addressing clinical challenges associated with poor visibility while significantly reducing procedural difficulties [15]. Second, the robotic arm’s multi-axis rotational capability facilitated optimal instrument positioning [27], demonstrating advantages in accessing the anatomically constrained maxillary second molar region. Notably, this case presented additional complexity due to an intact buccal bone plate, requiring a 9.6 mm resection depth at the MB root apex adjacent to the maxillary sinus. These factors present significant challenges to hand stability and tactile feedback if manual operation is adopted. The ATR system’s technological superiority is evident in its ability to maintain precision despite these anatomical challenges. As a system that provides autonomous rigid motion, ATR technology exceeds the physiological and experiential limitations of human operators, achieving levels of stability that are beyond manual capabilities [33]. The critical 3D spatial navigation was achieved through the ATR system’s integrated visual servo and force feedback mechanisms [13, 33]. These capabilities were crucial for implementing our pre-designed trajectory for maxillary sinus protection, which required exceptional precision to adhere to the principles of minimally invasive surgery. Although the DB root required freehand resection due to soft tissue obstruction, the robotic system provided essential positional references and enhanced visualization for accurate manual intervention.

To optimize optical tracking performance under the constraints of a specific patient’s position in this clinical scenario, we implemented an improved intraoral component designed for surgery in most posterior regions. The markers in previous case reports typically aligned perpendicular to the patient’s sagittal plane, which is suboptimal in this case due to the 65°−80° right-rotated head position. Such orientation of visual markers hinders the vertical tracking of infrared light, which may affect reaction accuracy and increase the need for additional intraoperative registration processes [25]. We adopted an improved intraoral component in this case, which aligns the visual marker parallel to the patient’s sagittal plane, thereby accommodating the specific head’s position during operation.

This case’s shortcoming lies in the failure to identify the MB2 apical foramen during surgery. The CBCT revealed diffuse calcification along the path of the MB2 canal, and the inherent anatomical constraints of the region still pose significant difficulties for searching the canal. Additionally, the most apical part of the main root canal, as well as the apical ramifications and lateral canals, are the most common areas of bacterial persistence [1, 19]. Successful resection and obturation of MB and DB root ends are expected to achieve effective infection control. This was corroborated by the satisfactory healing observed at the 6-month follow-up.

Intentional replantation is also an alternative approach for treating second molars with refractory periapical periodontitis [34, 35]. However, it is necessary to carefully consider the challenges in this case associated with minimally invasive tooth extraction [36], as well as the effects of granulation tissue curettage on the periodontal ligament for the tooth [34].

This case demonstrates the potential of the ATR system in mitigating the technique sensitivity during EMS on the maxillary second molar. This advancement extends ATR’s applications, while the autonomy and programmatic operation of robotics demonstrates the potential to achieve procedural standardization for second molar surgical protocols. We anticipate that the ATR system will hold greater promise for managing surgical procedures in challenging posterior regions, especially where manual operation is restricted. However, RA-EMS on the maxillary second molar imposes more stringent requirements for mouth opening and buccal mucosa traction, and novel miniaturized drilling systems remain to be developed. The precision of RA-EMS necessitates further clinical validation. It is also essential to simplify preoperative preparation and to incorporate machine learning. Furthermore, the additional costs related to 3D-printed accessories and instrumentation should be taken into account.

## Conclusion

We present a pioneer report evaluating the feasibility and efficacy of implementing an ATR system for the EMS on the maxillary second molar. RA-EMS offers an accurate, safe, and minimally invasive technique for osteotomy and apicoectomy in challenging anatomical areas within this case’s constraints. This advancement is anticipated to address challenges in the field of EMS that have proven difficult to resolve through manual operation technology, thereby ultimately improving and promoting patient-centered healthcare quality.

## Data Availability

No datasets were generated or analysed during the current study.

## Abbreviations

EMS: Endodontic microsurgery
ATR: Autonomous robotic
RCT: Root canal treatment
SN: Static navigation
DN: Dynamic navigation
RA-EMS: Robot-assisted endodontic microsurgery
MB2: Second mesiobuccal
MB: Mesiobuccal
DB: Distobuccal
CGF: Concentrated growth factors
